# Case Report: Olaparib combined with temozolomide and atezolizumab in a case of *BRCA2*-mutated small-cell transformation of lung adenocarcinoma

**DOI:** 10.3389/fonc.2026.1767999

**Published:** 2026-03-26

**Authors:** Meiyuan Liu, Pei Gao, Fu Liu, Deng Li, Yanqi Wang, Juan Yu

**Affiliations:** Department of Medical Oncology, Zhangjiajie City People’s Hospital, Zhangjiajie, Hunan, China

**Keywords:** atezolizumab, BRCA2 mutation, lung adenocarcinoma, olaparib, small-cell transformation, temozolomide

## Abstract

Small-cell transformation is a clinically resistant mechanism against epidermal growth factor receptor tyrosine kinase inhibitors (EGFR-TKIs) in lung adenocarcinoma (LUAD). It often leads to poor outcomes and limited treatment options. This case report describes an advanced case of LUAD with an EGFR exon 19 deletion and a *BRCA2* mutation. After failing several therapies, the disease progressed to a histologically confirmed small-cell lung cancer (SCLC) phenotype. In this case, treatment with olaparib (150 mg/day), combined with temozolomide (50 mg/day) and atezolizumab for two cycles, resulted in a partial tumor response. The patient experienced grade IV myelosuppression, consistent with a previous episode of severe haematologic toxicity during chemotherapy. This case highlights the potential value of combining olaparib, temozolomide, and immune checkpoint inhibition as a therapeutic strategy for *BRCA2*-mutated small-cell transformation arising from LUAD. However, significant haematologic adverse effects require careful monitoring.

## 1 Introduction

Lung cancer remains one of the most prevalent and lethal malignancies worldwide. In 2020, there were approximately 2.2 million newly diagnosed cases and 1.8 million deaths globally, making lung cancer the leading cause of cancer-related mortality for several consecutive years (1). From a histopathological perspective, non-small cell lung cancer (NSCLC) accounts for approximately 85% of all lung cancer cases, whereas small cell lung cancer (SCLC) represents about 15%. SCLC is characterized by high aggressiveness, rapid proliferation, and early metastasis (2). Among NSCLC subtypes, adenocarcinoma and squamous cell carcinoma are the most common. Notably, the incidence of adenocarcinoma has increased over the past decades, particularly among women and individuals who have never smoked (1). In recent years, the therapeutic landscape of lung cancer has undergone substantial transformation. In NSCLC, molecularly targeted therapies directed against driver gene alterations—including *EGFR, ALK, KRAS*, and *MET*—along with the introduction of immune checkpoint inhibitors (ICIs), have significantly prolonged patient survival (2, 3). For patients with extensive-stage SCLC, the combination of PD-(L)1 inhibitors with platinum–etoposide chemotherapy has become the standard first-line treatment. Although the overall survival benefit remains modest, this advancement marks the beginning of the immunotherapy era in SCLC management (2). Nevertheless, despite these therapeutic advances in both NSCLC and SCLC, acquired resistance and disease progression remain inevitable. This is particularly challenging when uncommon resistance mechanisms, such as histologic transformation, emerge, as treatment options in such scenarios are extremely limited.

Epidermal growth factor receptor tyrosine kinase inhibitors (EGFR-TKIs) have significantly improved patients’ survival with advanced lung adenocarcinoma (LUAD) harboring *EGFR* mutations. Despite this progress, acquired resistance to these agents is almost inevitable. Resistance usually develops within 9–14 months of treatment, with the secondary *T790M* mutation responsible for approximately 60% of cases (4–8). More recently, transformation to SCLC has been recognized as a less common but clinically significant mechanism of resistance. Reports suggest that SCLC transformation occurs in approximately 3-14% of *EGFR*-mutant adenocarcinomas progressing on EGFR-TKIs (7, 9, 10). This transformation is associated with poor prognosis, and therapeutic options remain limited, especially in the presence of *BRCA2* mutations. Studies show that poly (ADP-ribose) polymerase (PARP) inhibitors, particularly when combined with DNA-damaging agents or immune checkpoint blockade, may provide clinical benefit in SCLC. To date, no cases have documented the use of a triple-combination regimen comprising a PARP inhibitor, a DNA-damaging agent, and an ICI in patients with *BRCA2*-mutated SCLC arising from LUAD. This report describes the first known case of *EGFR*-mutated LUAD with SCLC transformation carrying a *BRCA2* mutation, treated with olaparib, temozolomide, and atezolizumab. Clinical outcomes and safety observations are presented, providing preliminary evidence to guide the development of targeted therapeutic strategies for this rare and aggressive disease phenotype.

## 2 Case presentation

**Figure 1:**
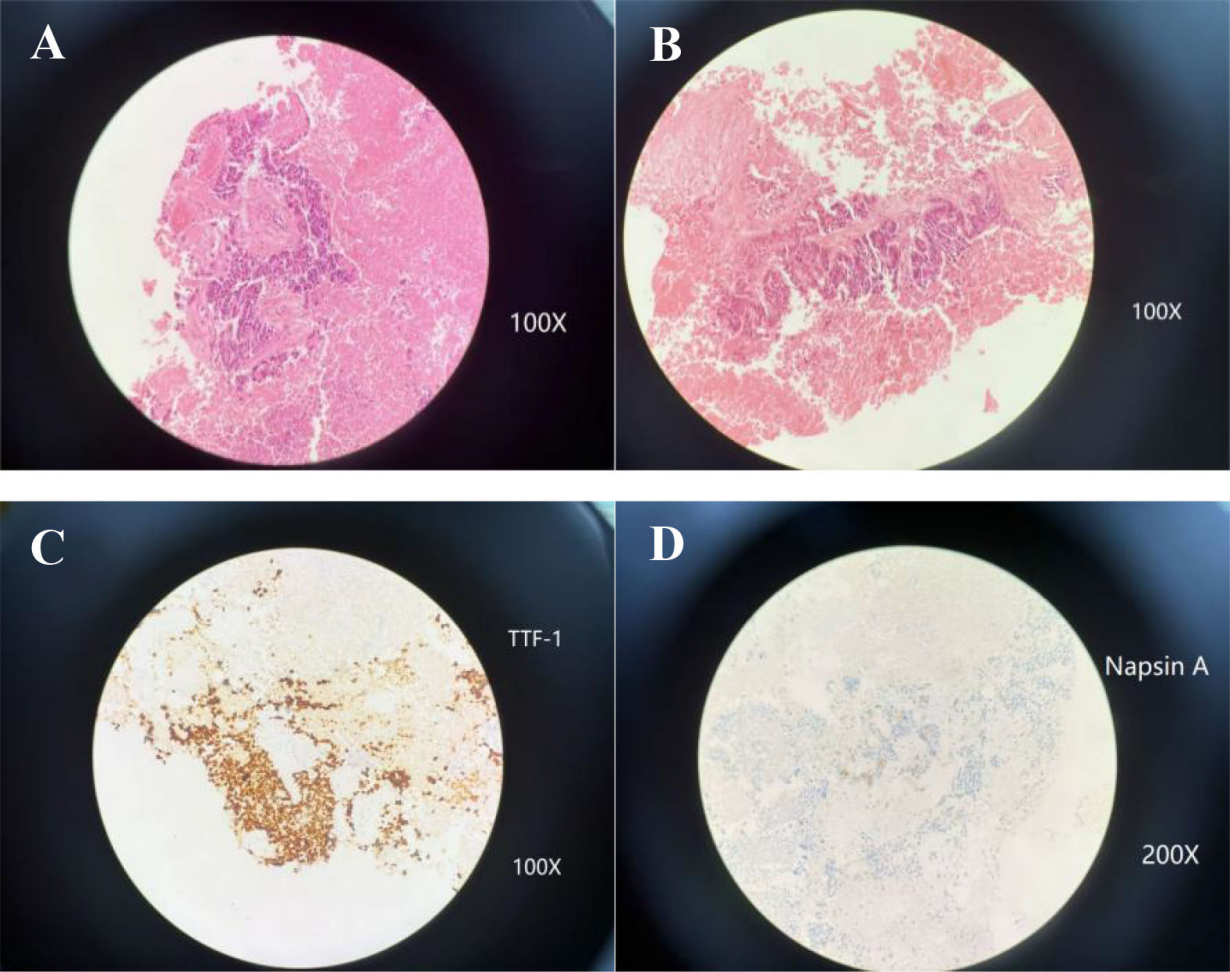
Initial liver biopsy showed a poorly differentiated adenocarcinoma with focal squamous features, consistent with a primary lung tumor. **(A, B)** Histopathological examination reveals a highly disordered cellular structure, with areas forming irregular gland-like structures alongside areas exhibiting squamous differentiation. Neoplastic cells have hyperchromatic nuclei, distinct nucleoli, significant nuclear pleomorphism, and occasional spindle-shaped forms [H&E stain; **(A)** 100x; **(B)** 100x]. **(C)** Immunohistochemical staining for thyroid transcription factor-1 (TTF-1) shows strong nuclear reactivity, supporting a pulmonary origin (IHC 100x). **(D)** Napsin-A displays focal cytoplasmic staining, further confirming the diagnosis of lung adenocarcinoma (IHC 200x).

**Figure 2:**
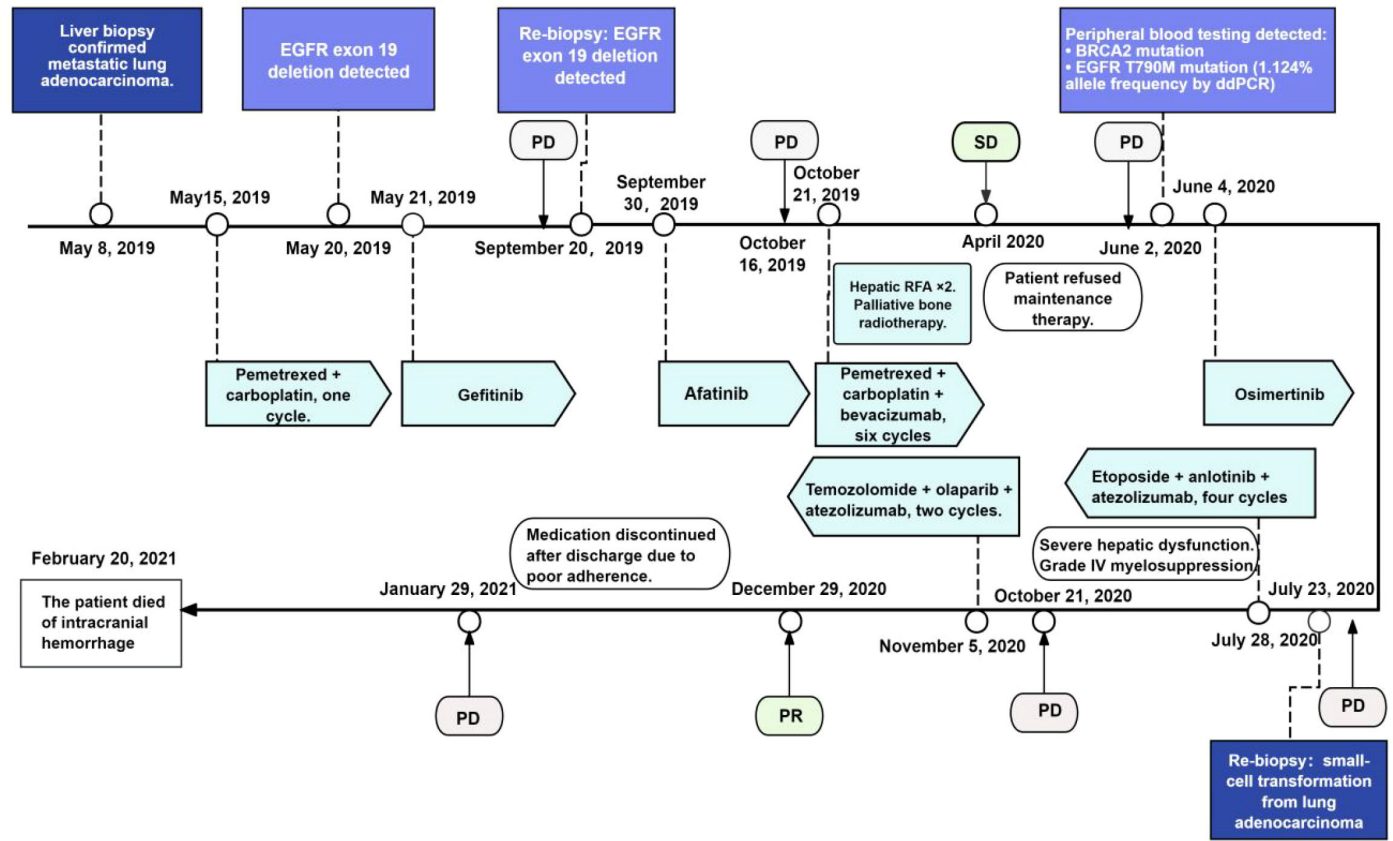
Timeline of diagnosis, treatments, and response assessments. PD, progressive disease; SD, stable disease; PR, partial disease, EGFR, epidermal growth factor receptor; ddPCR, droplet digital PCR.

A 47-year-old woman with no history of smoking presented on 5 May 2019, reporting a two-month history of upper abdominal discomfort and a recently identified hepatic mass, discovered four days before presentation. The patient’s past medical and family histories were unremarkable. On physical examination, no superficial lymphadenopathy was detected. Mild tenderness was noted in the right upper abdominal quadrant without rebound pain. A positron emission tomography–computed tomography (PET-CT) scan revealed a soft-tissue lesion in the posterior segment of the left upper lung apex, showing intense fluorodeoxyglucose uptake, suggestive of a primary pulmonary malignancy. Other findings included multiple metastatic lesions in the liver and osseous involvement, specifically affecting the T9 vertebra and the left ilium. On 8 May 2019, an ultrasound-guided biopsy of the hepatic lesion was performed. Histopathological examination revealed poorly differentiated carcinoma consistent with adenocarcinoma (**Figure 1**). Immunohistochemical analysis showed focal squamous differentiation and suggested a primary pulmonary origin. Tumor cells tested positive for CK7, CK19, Villin, TTF-1, and showed focal positivity for Napsin A and P40, with a Ki-67 proliferative index of approximately 70%. Staining for CK20, AFP, CD10, CD34, WT-1, PAX-8, GATA3, Hep-Par-1, ALK, Synaptophysin, and Chromogranin A was negative, while CDX-2 showed weak and focal positivity. Combining these findings with PET-CT imaging, a diagnosis of primary peripheral lung adenocarcinoma of the left upper lobe (cT1N0M1, stage IV) with extensive hepatic and skeletal metastases was established. The patient’s subsequent diagnostic and therapeutic course is illustrated in **Figure 2**.

The patient initiated systemic therapy with a single cycle of pemetrexed (600 mg) and carboplatin (450 mg) on 15 May 2019. On 20 May 2019, next-generation sequencing (Suzhou Konuodi) identified TP53 (80.42%), PIK3CA (22.59%), and an EGFR exon 19 deletion (48.07%), with a tumor mutational burden (TMB) of 9 mutations/Mb. Based on these molecular findings, targeted therapy with gefitinib (250 mg/day) was started. The patient tolerated the treatment well, and follow-up computed tomography (CT) scans were carried out bi-monthly. Radiological assessments according to RECIST v1.1 indicated stable disease (SD). However, a CT scan on 20 September 2019 showed growth of the pulmonary nodule in the left upper lobe, along with a slight increase in several hepatic metastatic lesions, findings that were highly indicative of newly developed therapeutic resistance. A second liver biopsy was performed on 21 September 2019, and targeted analysis using an eight-gene lung cancer panel confirmed the presence of an EGFR exon 19 deletion (34.71%). Based on these findings, the treatment plan was adjusted, and afatinib (40 mg/day) was started on 30 September 2019. By 16 October 2019, the patient reported a recurrence of liver-related pain. Follow-up CT imaging showed further progression of hepatic metastatic involvement, and the disease was classified as progressive disease (PD). Between 21 October 2019 and 26 April 2020, the patient received six cycles of a combination regimen comprising pemetrexed disodium (700 mg) and carboplatin (500 mg), with bevacizumab (400 mg) as a targeted agent. During this treatment period, two radiofrequency ablation procedures were performed on hepatic tumors, and palliative radiotherapy was delivered to metastatic sites in the left ilium and at T9 (30 Gy in 10 fractions for each area). Follow-up CT imaging in April 2020 showed SD. Maintenance therapy with pemetrexed and bevacizumab was suggested but declined by the patient. On 2 June 2020, CT scans showed multiple new osseous metastases involving the T1 vertebra, multiple lumbar vertebrae, bilateral ischial tuberosities, and the left pubic bone. Post-ablation changes were observed in previously treated hepatic lesions, while additional low-density hepatic nodules indicated persistent metastatic disease, consistent with PD. Peripheral blood next-generation sequencing detected *EGFR* exon 19 deletion (36.8%) and *PIK3CA* mutation (9.86%), with a high TMB of 20.714 mutations/Mb. A *BRCA2* mutation (6.22%) was also identified among variants of uncertain significance. Droplet digital PCR showed a *T790M* mutation at 1.124%, prompting the initiation of osimertinib (80 mg/day) on 4 June 2020. A CT scan of the head, chest, and abdomen dated 23 July 2020 showed the following findings: (1) a pulmonary nodule in the left upper lobe that has remained largely unchanged from previous studies; (2) stable calcified nodular densities in both the right upper and lower lobes; (3) post-ablation cavities corresponding to previously treated hepatic metastatic lesions that have decreased in size, along with a significant increase in the number of remaining low-attenuation metastatic nodules scattered throughout the residual liver parenchyma; (4) the presence of a benign-appearing cyst within the left kidney; (5) no significant intracranial abnormalities detected on head CT imaging; and (6) multiple metastatic bone lesions involving the thoracic vertebrae, lumbar spine, and pelvic bones, generally similar to earlier imaging assessments. MRI of the liver revealed: (1) a heterogeneous lesion located in the posterior segment of the right hepatic lobe, raising concern for hepatocellular carcinoma with features indicative of cystic degeneration and necrosis; (2) various solid hepatic nodules distributed throughout the liver, highly suggestive of metastatic deposits, with several larger lesions considered suitable for tissue sampling; (3) no significant findings involving the spleen, pancreas, or bilateral kidneys; and (4) an absence of discrete masses within either adrenal gland region. These radiological observations were consistent with PD.

**Figure 3:**
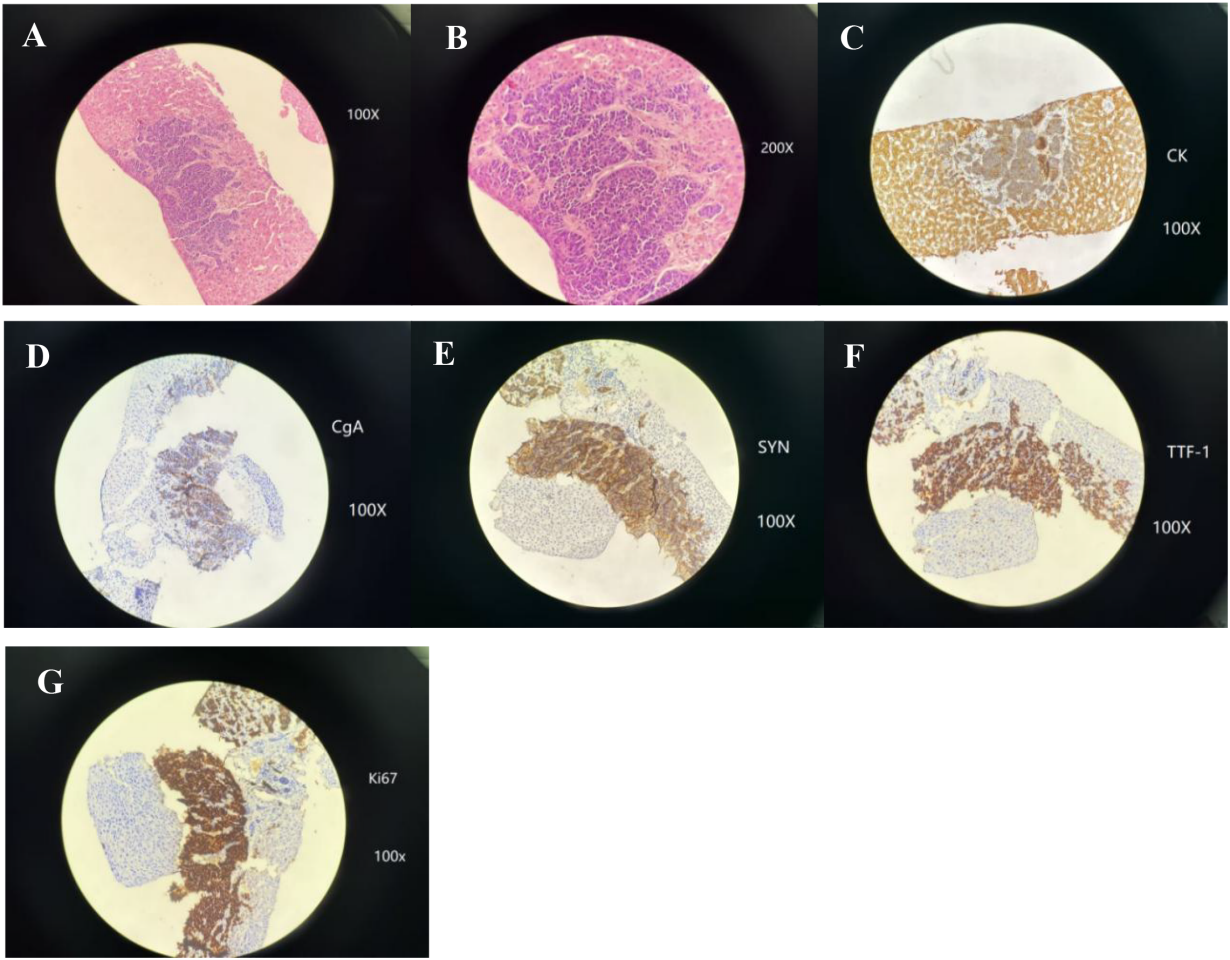
The second liver biopsy confirmed transformation into small-cell carcinoma, again indicative of pulmonary origin. **(A, B)** Microscopic sections show tightly packed malignant cells arranged in compact nests and sheets. These cells are small, round to oval, with high nuclear-to-cytoplasmic ratios, finely stippled “salt-and-pepper” chromatin, inconspicuous nucleoli, and a complete absence of glandular or squamous differentiation [H&E stain, **(A)** 100x, **(B)** 200x]. **(C)** Cytokeratin (CK) immunostaining highlights characteristic paranuclear dot-like cytoplasmic positivity (IHC 100x). **(D)** Chromogranin A (CgA) exhibits partial cytoplasmic immunoreactivity (IHC 100x). **(E)** Synaptophysin (Syn) shows diffuse cytoplasmic positivity throughout the tumor (IHC 100x). **(F)** Thyroid transcription factor-1 (TTF-1) again reveals strong nuclear staining, supporting a pulmonary neuroendocrine phenotype (IHC 100x). **(G)** Ki-67 proliferative index approaches 90%, indicating exceptionally rapid cellular proliferation (IHC 100x).

On 24 July 2020, a further liver biopsy was performed for additional diagnostic clarification. Histopathological examination (H&E staining) and immunohistochemical analysis revealed features of small-cell carcinoma. Considering the patient’s previous clinical and diagnostic history, the pathological findings strongly indicated a pulmonary primary origin, supporting the diagnosis of SCLC (**Figure 3**).

Immunohistochemical analysis showed strong expression of LCK, TTF-1, cytokeratin (CK), CK19, Glypican-3, synaptophysin, Villin, and CD56, along with an increased proliferative rate, with Ki-67 nearing 90%. The tumor was negative for P40, CK20, P63, CK7, CK5/6, and Hep-Par-1, while chromogranin A showed only focal reactivity. To evaluate disease burden and treatment response following small-cell transformation, serum tumor markers were routinely measured during outpatient follow-up visits or hospitalization. These assessments were not conducted at strictly predefined time intervals; rather, they were performed based on clinical need, including evaluation of disease progression or therapeutic response (see **Table 1**). Tumor markers were used only as supplementary indicators, while treatment response was primarily evaluated based on imaging findings.

**Table 1:** Dynamic monitoring of tumor markers after small-cell transformation.

Date	CEA (ng/mL)	NSE (ng/mL)	CA125 (U/mL)	CA19-9 (U/mL)	SCC (ng/mL)
2020-07-18	299	>300*	812	–	1.5
2020-08-01	938	>300*	2275	126	1.47
2020-10-14	>1000*	74	1557	255	2.25
2020-11-24	>1000*	11.3	386	44.9	1.9
2020-12-30	952	20	333	73	0.63
2021-01-30	>1000*	49	1296	–	0.87
2021-02-20	>1000*	>300*	2141	–	1.29

*Values exceeding the upper detection limit of the assay (right-censored values).

– Not available.

**Figure 4:**
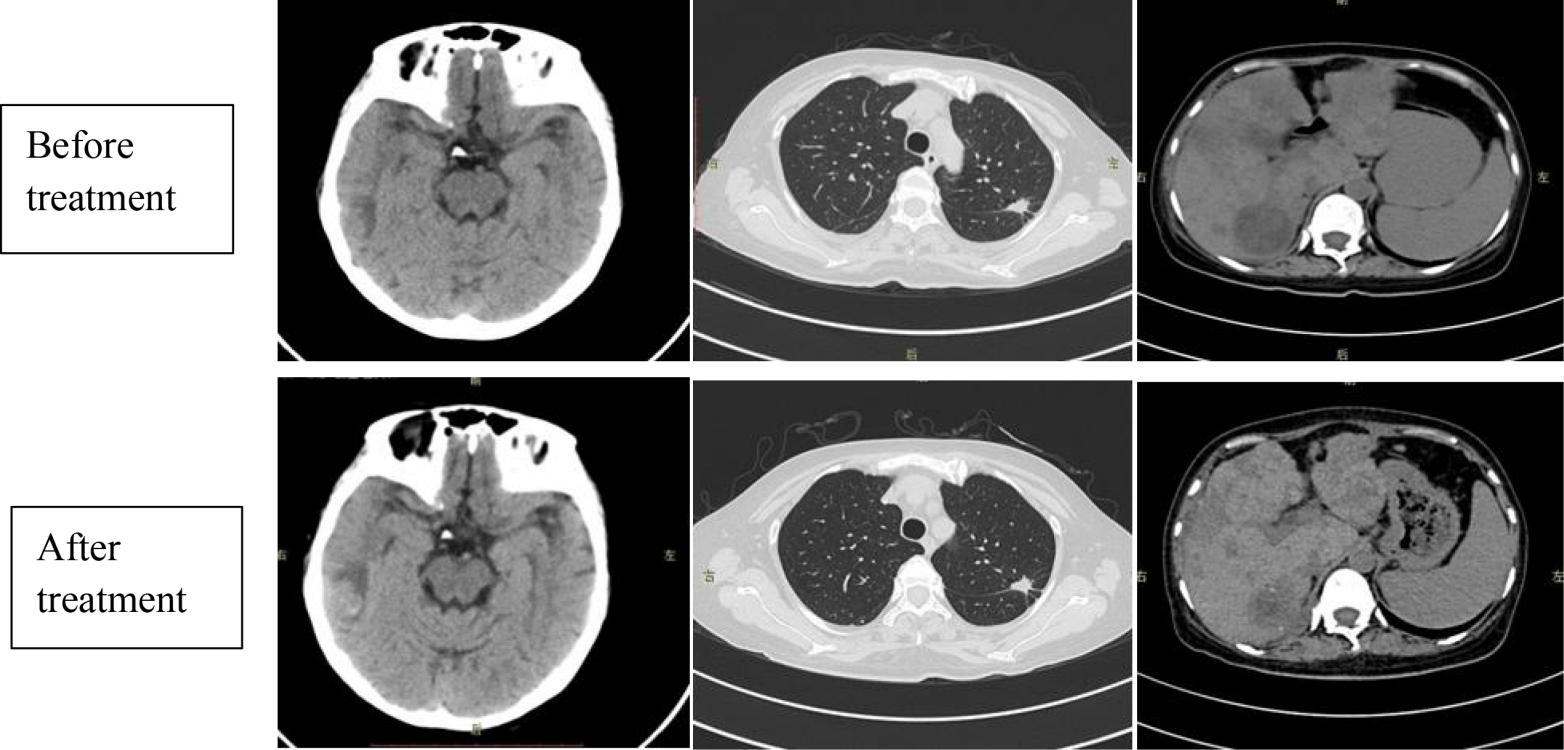
Comparative CT imaging before and after two cycles of treatment with olaparib, temozolomide, and atezolizumab.

A biochemical analysis on 27 July 2020 indicated significant deterioration of hepatic function, with bilirubin and transaminase levels steadily rising, consistent with rapidly progressing tumor burden. As the patient’s hepatic capacity continued to decline and her clinical condition rendered her unable to tolerate conventional intravenous chemotherapy, a modified treatment plan was initiated on 28 July 2020. The regimen included oral etoposide (50 mg, Days 1–5), anlotinib (8 mg, Days 1-14), and atezolizumab (1200 mg on Day 1, repeated every 21 days). On 31 July 2020, the patient presented with abdominal swelling, severe fatigue, decreased appetite, and progressive jaundice. Laboratory tests at that time revealed thrombocytopenia, mild anemia, and significant hepatic impairment. During her hospital stay, she received hepatoprotective medications, agents to alleviate cholestasis, and treatments to increase platelet production. Periodic albumin infusions were also administered to address hypoalbuminemia, alongside comprehensive nutritional supplementation. These interventions resulted in a clear improvement in liver enzyme levels compared to initial results. However, on 14 August 2020, she developed a fever. Blood cultures identified *Escherichia coli*, leading to targeted antibiotic treatment based on susceptibility results. Repeat blood tests demonstrated grade IV myelosuppression involving all three hematopoietic lineages. She was treated with two therapeutic doses of single-donor platelet transfusion and two units of packed red blood cells, resulting in clinical improvement. Between 25 August and 6 October 2020, three cycles of therapy using oral etoposide, anlotinib, and atezolizumab were administered. On 13 October 2020, the patient was readmitted with a low-grade fever. Laboratory tests showed neutrophilia and moderate anemia, along with significantly elevated tumor markers (see **Table 1**). CT scans on 21 October 2020 revealed stable pulmonary nodules in the left upper lobe, resolution of inflammatory changes in the left lower lobe, largely unchanged osseous metastases, and persistent hepatic lesions. A new low-density lesion at the right temporo-occipital junction raised concerns of intracranial metastasis. Radiological assessment indicated PD. On 5 November 2020, the patient started a therapeutic regimen that included atezolizumab (1200 mg on Day 1), continuous oral temozolomide (50 mg/day), and olaparib (150 mg/day). By 23 November 2020, she was readmitted to the hospital due to severe fatigue. Hematologic analysis showed grade IV pancytopenia affecting all hematopoietic lineages, along with biochemical confirmation of hepatic impairment. During this hospitalization, the patient received supportive transfusions, including 4 units of packed red blood cells and two doses of single-donor platelets, along with medications to support liver function. Both liver biochemical markers and marrow suppression showed significant improvement after supportive care. A second cycle of the same therapeutic regimen, atezolizumab (1200 mg intravenously), temozolomide (50 mg/day), and olaparib (150 mg/day), was administered on 12 December 2020. The patient returned for reassessment on 29 December 2020. Laboratory testing at that time indicated grade I myelosuppression, manifesting as leukopenia along with mild anemia. Serum tumor biomarkers exhibited substantial decreases compared to previous measurements: CEA declined from >1000 ng/mL to 952 ng/mL, NSE decreased from 74 ng/mL to 20 ng/mL, CA125 dropped from 1557 U/mL to 333 U/mL, CA19–9 fell from 255 U/mL to 73 U/mL, and SCC was reduced from 2.25 ng/mL to 0.63 ng/mL (see **Table 1**). A follow-up CT scan reading, compared with previous imaging from 21 October 2020, showed several important findings: (1) The left upper lobe pulmonary nodule mainly remained unchanged, and inflammatory changes previously noted within the left lower lobe had resolved; several skeletal metastatic lesions appeared marginally enlarged. (2) Multiple liver metastatic deposits had decreased in dimension, with the largest lesion contracting from 46 mm to 29 mm; mild dilatation of the intrahepatic biliary tree and localized narrowing of the intrahepatic inferior vena cava were also observed. (3) A low-density lesion with adjacent nodularity at the right temporo-occipital junction showed no change over time (**Figure 4**).

The radiologic findings and reductions in biochemical tumor markers supported an overall assessment of partial response (PR). Despite observable clinical benefit, the patient discontinued systemic therapy after discharge due to poor adherence. A CT scan on 29 January 2021 showed disease progression, with enlargement of pulmonary nodules in both upper lobes and worsening metastatic involvement of the liver, indicating PD. On 20 February 2021, the patient died from an intracranial hemorrhage caused by grade IV bone marrow failure resulting from chemotherapy-related toxicity.

## 3 Discussion

The use of EGFR-targeted tyrosine kinase inhibitors has significantly extended survival in patients with NSCLC. Despite these therapeutic advancements, most patients eventually face therapeutic failure due to the emergence of resistance, which leads to renewed tumor progression. Previous studies have identified multiple biological processes that contribute to EGFR-TKI resistance, including the acquisition of additional *EGFR* modifications, amplification of *MET* or *HER2*, activation of alternative signaling pathways, mutations in downstream effectors, and histological evolution to SCLC, among others (11–16). Although the incidence of SCLC transformation is lower than that of classical target-mutation-mediated resistance, it is associated with greater clinical aggressiveness and more limited therapeutic options, making it a particularly challenging resistance pattern.

Importantly, treatment-induced SCLC transformation differs significantly from primary SCLC in both molecular characteristics and clinical behavior. At the molecular level, transformed SCLC typically retains the original driver mutations of lung adenocarcinoma, such as *EGFR* mutations, whereas primary SCLC rarely harbors such actionable genetic alterations (9, 17). This finding suggests that transformed SCLC arises from clonal evolution of the original tumor rather than representing a newly developed, independent malignancy. Furthermore, although concurrent loss of *RB1* and *TP53* is common in both transformed and primary SCLC, transformed SCLC often exhibits a clonal evolutionary trajectory and genomic instability patterns shared with the original adenocarcinoma (17–19). Clonal evolution analyses by Lee et al. demonstrated that *RB1* and *TP53* alterations were already present in *EGFR*-mutant adenocarcinoma prior to SCLC transformation, indicating a pre-existing potential for neuroendocrine differentiation (17). Clinically, compared with *de-novo* SCLC, transformed SCLC generally shows a shorter duration of response to standard platinum-etoposide chemotherapy and is associated with a poorer overall prognosis (9). Marcoux et al. further reported that patients with transformed SCLC had significantly shorter overall survival than those in conventional SCLC cohorts (9). Regarding immunotherapy response, some studies suggest that transformed SCLC may exhibit lower PD-L1 expression and a distinct tumor immune microenvironment compared with primary SCLC, potentially affecting responsiveness to immune checkpoint inhibitors (20). Taken together, transformed SCLC appears to represent a”treatment-induced resistant state”rather than a traditional primary neuroendocrine carcinoma.

A deeper understanding of the molecular mechanisms underlying SCLC transformation is essential for developing personalized therapeutic strategies and identifying novel potential targets. Recent studies suggest that type II alveolar epithelial cells may serve as common progenitors for both adenocarcinoma and SCLC. Under selective pressure from EGFR-TKI therapy, a subset of tumor cells that would normally differentiate into an adenocarcinoma lineage might instead undergo phenotypic reprogramming towards an SCLC identity. Another hypothesis proposes that some patients have mixed histological subtypes at diagnosis; in these cases, prolonged TKI exposure may selectively suppress the adenocarcinoma component while allowing the neuroendocrine (SCLC) component to proliferate, ultimately leading to acquired drug resistance (21, 22). Growing molecular evidence indicates that *RB1* and *TP53* are key factors in the progression of *EGFR*-mutant adenocarcinoma to SCLC. Simultaneous loss of *RB1* and *TP53* expression promotes the development of a neuroendocrine phenotype from adenocarcinoma cells (17–20, 22, 23). The joint inactivation of these two tumor-suppressor genes has been suggested as a potential molecular marker for predicting imminent SCLC transformation. Enhancer of zeste homolog 2 (EZH2), a key epigenetic regulator of chromatin, has also been associated with oncogenesis. Multiple experimental findings indicate that *EZH2* may be a promising target for intervention, capable of reducing or reversing neuroendocrine transdifferentiation (NET) (24, 25). Further resistance-related pathways have been identified. Activation of Notch-1 signaling has been associated with the induction of epithelial–mesenchymal transition (EMT) and the development of gefitinib resistance (26). The Notch pathway acts as a key regulatory hub overseeing neuroendocrine plasticity in NSCLC, allowing tumor cells to adopt SCLC characteristics after initial exposure to targeted therapy and thus promoting therapeutic resistance to EGFR-TKIs (27). For tumors that have transitioned from adenocarcinoma to SCLC, the NOTCH–ASCL1 regulatory axis has been suggested as a potential therapeutic vulnerability. Despite these emerging insights, the biological circuitry regulatory transition from adenocarcinoma to SCLC remains only partly understood. Further research is needed to fully define the processes driving this aggressive phenotypic change.

*BRCA1* and *BRCA2* function as pivotal tumor suppressor genes involved in homologous recombination repair and transcriptional regulation. Pathogenic changes in these genes significantly contribute to the development of various malignancies. In a previous study, Yan et al. (28) evaluated the prevalence of *BRCA* variants among 462 individuals with lung cancer from south-central China using next-generation sequencing. Their findings showed mutation frequencies of 4.3% for *BRCA1* and 6.5% for *BRCA2*, which align closely with global data. Interestingly, the highest rate of *BRCA* alterations (18.2%) was observed in individuals aged 40 to 49 years. In another large-scale study, Hu et al. (29) examined 6,220 Chinese lung cancer cases and found germline *BRCA* mutations in 1.03% of the population, with *BRCA2* variants comprising 76.3% of those identified. The presence of a *BRCA2* alteration in the current 47-year-old patient aligns well with the demographic patterns reported in these studies. Comprehensive proteomic and transcriptomic profiling has shown that PARP1 expression is significantly increased in SCLC compared to other pulmonary tumor subtypes (30). Although pathogenic BRCA1/2 variants are rare in SCLC (31, 32), the loss of *BRCA* function provides a biologically rational target for PARP inhibition. Experimental research indicates that SCLC cell lines remain sensitive to PARP blockade even without *BRCA* mutations (30), suggesting that deficiencies in other DNA repair pathways may generate similar vulnerability. PARP inhibitors are already well established in treating *BRCA*-mutated ovarian and breast cancers; however, clinical data on their therapeutic effectiveness in *BRCA2*-mutated SCLC are very limited. For cases of SCLC arising from phenotypic transformation of lung adenocarcinoma, treatment options are currently limited beyond conventional regimens for *de novo* SCLC. After the emergence of this transformed phenotype, platinum-etoposide chemotherapy yields an objective response rate of approximately 54%. However, the durability of these responses is poor, with a median progression-free survival (mPFS) of approximately 3.4 months (9). Most cytotoxic and targeted anticancer agents function through mechanisms closely related to DNA injury and repair. Extensive preclinical research across various solid tumor models has shown that combining PARP inhibitors with the DNA-damaging agent temozolomide produces a synergistic antitumor effect, highlighting a promising therapeutic pathway (33).

Temozolomide exerts its cytotoxic effect by adding methyl groups to specific nucleotide bases, generating DNA lesions usually repaired by the O^6^-methylguanine-DNA methyltransferase (MGMT), the mismatch repair (MMR) pathway, and the base excision repair (BER) machinery. Inhibition of PARP activity disrupts BER, increasing tumor cells’ sensitivity to temozolomide-induced DNA damage (34, 35). A phase I/II single-arm study of the combination of olaparib and temozolomide in previously treated SCLC reported an objective response rate (ORR) of 41.7%, with mPFS of 4.2 months and median overall survival (mOS) of 8.5 months. These outcomes exceeded those observed in several recent second- and third-line therapeutic studies for SCLC (36). This study provided the earliest clinical evidence supporting the activity of the olaparib–temozolomide combination in relapsed SCLC. In another phase II trial comparing temozolomide plus veliparib versus temozolomide plus placebo in patients with sensitive or refractory relapsed SCLC, the veliparib cohort showed a significantly higher ORR. Similarly, expression of Schlafen 11 (SLFN11) was associated with prolonged progression-free and overall survival among individuals treated with the veliparib–temozolomide combination, although increased rates of grade 3–4 neutropenia and thrombocytopenia were observed in the experimental arm (37). These findings reinforce the role of SLFN11 as a predictive biomarker for identifying SCLC subpopulations most likely to benefit from PARP1 blockade (37–40). Current evidence demonstrates that combining PARP inhibitors with temozolomide improves antitumor activity through a synthetic lethality mechanism.

Atezolizumab, an anti-programmed death ligand 1 (PD-L1) monoclonal antibody, counteracts immune suppression by inhibiting the PD-1/PD-L1 checkpoint interaction, thus reactivating cytotoxic T-cell responses. The clinical benefit of immunotherapy in SCLC appears to be largely confined to first-line treatment in combination with platinum-based chemotherapy (41). The olaparib–temozolomide combination increases the burden of DNA damage by synergistically impairing DNA repair pathways. Enhanced genomic injury can activate innate immune signaling cascades, including the STING pathway, which may potentiate the antitumor effects of atezolizumab (42, 43). Furthermore, data suggest that PARP inhibition may boost the activity of ICIs by inducing PD-L1 upregulation and facilitating T-cell infiltration within the tumor microenvironment (44). However, evidence regarding the clinical benefit of combining PARP inhibitors with immunotherapy in SCLC remains limited, particularly from prospective randomized trials. The recently published S1929 randomized phase II study evaluated maintenance therapy with the PARP inhibitor talazoparib plus atezolizumab versus atezolizumab alone in 106 patients with SLFN11-positive extensive-stage SCLC. The combination arm demonstrated a modest prolongation in median progression-free survival (PFS) compared with monotherapy (2.9 vs. 2.4 months; HR = 0.66), representing a gain of only 0.5 months. However, no overall survival (OS) benefit was observed (HR = 0.98). Notably, grade ≥3 hematologic adverse events were significantly more frequent in the combination group (50% vs. 4%) (45). These findings suggest that even in a biomarker-selected population of SLFN11-positive SCLC, the clinical benefit of PARP inhibitors combined with immunotherapy remains modest and has not translated into a clear survival advantage. Current evidence is therefore insufficient to support the routine use of this combination strategy in SCLC. In the context of limited high-level evidence, the clinical value of combining PARP inhibitors with immunotherapy—and whether adding immunotherapy to a backbone of PARP inhibitors plus DNA-damaging agents may provide synergistic benefit—requires further validation.

Previous studies have demonstrated that PARP inhibitors combined with temozolomide exhibit antitumor activity in relapsed SCLC (36). In the present case, after two cycles of olaparib plus temozolomide and atezolizumab, the patient experienced a marked decline in tumor markers and significant shrinkage of multiple intrahepatic metastases. The observed response may have been primarily attributable to the synergistic cytotoxic interaction between the PARP inhibitor and temozolomide, while the independent contribution of immunotherapy cannot be clearly delineated. Importantly, at the time of confirmed SCLC transformation, the patient experienced rapid deterioration of liver function and was deemed unable to tolerate standard intravenous chemotherapy. Therefore, the selected regimen represented a feasible alternative under the prevailing clinical constraints. The use of immunotherapy in this case extended beyond the established first-line platinum-based chemoimmunotherapy framework and the standard second-line option of topotecan. The treatment decision was not based on established superiority but rather constituted an exploratory attempt in the context of a unique molecular background (*BRCA2* mutation with SCLC transformation) and significant clinical limitations. Unfortunately, due to poor performance status and severe post-treatment myelosuppression, the patient was unable to complete subsequent therapy and ultimately died from intracranial hemorrhage secondary to grade IV chemotherapy-induced bone marrow suppression. This outcome highlights the substantial hematologic toxicity risk associated with such regimens in heavily pretreated patients with compromised bone marrow reserve and organ dysfunction. Although the patient elected to continue antitumor therapy after being fully informed of the potential risks and limited expected benefit, retrospective reflection suggests that best supportive care might have represented a safer alternative.

This study has several limitations. First, this is a retrospective single-case report without a control group or long-term follow-up, which limits the generalizability of the findings. Because olaparib, temozolomide, and atezolizumab were administered concurrently, the relative contribution of each agent or combination to the observed therapeutic effect cannot be determined, and attribution of efficacy remains uncertain. Second, tumor marker assessments were retrospectively collected at non-uniform intervals, and some values were truncated at upper detection limits; therefore, these data serve only as supportive references consistent with imaging findings. Third, deviation from standard first-line platinum-based chemotherapy and second-line topotecan may reduce the comparability and broader applicability of the results. Fourth, due to the retrospective nature of the case, original pathological images were derived from archived clinical materials; scale bars were not preserved during digitization, and the original slides could not be rescanned. Nevertheless, magnification levels are clearly indicated in the figure legends, and key morphological and immunohistochemical features essential for diagnosis are adequately demonstrated. Finally, as the patient passed away several years ago, subjective treatment experiences were not systematically documented in the original medical records. In addition, the family members were unable to fully recall specific details of the clinical course. Therefore, we were unable to provide a”Patient Perspective”section. The absence of complete patient-perspective information may limit a comprehensive assessment of the balance between treatment benefits and potential risks.

## 4 Conclusions

Treatment options for SCLC transformed from lung adenocarcinoma remain limited, and responses to standard chemotherapy are often short-lived. This case suggests that the combination of olaparib, temozolomide, and an immune checkpoint inhibitor may represent a potential therapeutic option for patients with SCLC transformation harboring a *BRCA2* mutation. However, as this observation is based on a single case, the observed response should be interpreted as a time-associated clinical change rather than definitive evidence of efficacy and should be regarded as hypothesis-generating. Whether the addition of immunotherapy to a regimen combining PARP inhibition and DNA-damaging agents confers true synergistic benefit in SCLC remains to be determined. Larger prospective studies are warranted to validate the efficacy and safety of this approach and to identify the patient populations most likely to benefit.

## Data Availability

The original contributions presented in the study are included in the article/supplementary material. Further inquiries can be directed to the corresponding author.
